# The New York Medical Record

**Published:** 1888-10

**Authors:** 


The New York *Medical Record*, with its usual enterprise, devoted its entire edition of September 22, 1888, excepting one page, to a report of the proceedings of the Congress ot American Physicians and Surgeons and of the special societies that met in conjunction with it, in Washington, September 18th, 19th and 20th. This is a valuable index to the proceedings, and will undoubtedly be in large demand outside of its regular subscription list.

